# A genetically encoded fluorescent temperature sensor derived from the photoactive Orange Carotenoid Protein

**DOI:** 10.1038/s41598-019-45421-7

**Published:** 2019-06-20

**Authors:** Eugene G. Maksimov, Igor A. Yaroshevich, Georgy V. Tsoraev, Nikolai N. Sluchanko, Ekaterina A. Slutskaya, Olga G. Shamborant, Tatiana V. Bobik, Thomas Friedrich, Alexey V. Stepanov

**Affiliations:** 1 Lomonosov Moscow State University, Department of Biophysics, Faculty of Biology, 119991 Moscow, Russia; 2 A.N. Bach Institute of Biochemistry, Federal Research Center of Biotechnology of the Russian Academy of Sciences, 119071 Moscow, Russia; 3 M.M. Shemyakin and Yu.A. Ovchinnikov Institute of Bioorganic Chemistry, Russian Academy of Sciences, Miklukho-Maklaya Str., 16/10, Moscow, 117997 Russia; 4 Technical University of Berlin, Institute of Chemistry PC 14, Straße des 17. Juni 135, D-10623 Berlin, Germany

**Keywords:** Biological fluorescence, Biophysical methods

## Abstract

The heterogeneity of metabolic reactions leads to a non-uniform distribution of temperature in different parts of the living cell. The demand to study normal functioning and pathological abnormalities of cellular processes requires the development of new visualization methods. Previously, we have shown that the 35-kDa photoswitchable Orange Carotenoid Protein (OCP) has a strong temperature dependency of photoconversion rates, and its tertiary structure undergoes significant structural rearrangements upon photoactivation, which makes this protein a nano-sized temperature sensor. However, the determination of OCP conversion rates requires measurements of carotenoid absorption, which is not suitable for microscopy. In order to solve this problem, we fused green and red fluorescent proteins (TagGFP and TagRFP) to the structure of OCP, producing photoactive chimeras. In such chimeras, electronic excitation of the fluorescent protein is effectively quenched by the carotenoid in OCP. Photoactivation of OCP-based chimeras triggers rearrangements of complex geometry, permitting measurements of the conversion rates by monitoring changes of fluorescence intensity. This approach allowed us to determine the local temperature of the microenvironment. Future directions to improve the OCP-based sensor are discussed.

## Introduction

Intracellular temperature is crucial for the functional and metabolic activity of the cell since it determines the rates of all chemical reactions. First attempts to develop systems for detecting intracellular temperature appeared quite recently and attracted widespread interest in the scientific community^1–5^ (reviewed in^6^). Interest in intracellular temperature sensors is largely explained by prospects of using such systems to visualize various metabolic processes and to investigate disorders leading to the development of pathological conditions. Regardless of the physical principle underlying their functioning (ratiometric, rotometric or other), most of the existing intracellular temperature sensors are synthetic hybrid systems based on organic fluorescent dyes^1,4,7–9^. A common drawback of such systems is the complexity of practical application associated with the need to introduce synthetic dyes into the cell, as well as the complexity of data interpretation. For example, in the work of Okabe *et al*., the intracellular temperature was mapped based on the fluorescence lifetime distribution of N-(2-[(7-N,N-dimethylaminosulfonyl)-2,1,3-benzoxadiazol-4-yl](methyl)amino)ethyl-N-methylacrylamide (DBD-AA) as a part of a polymeric construction^1^. Although the authors developed a robust and sensitive probe with a temperature resolution of better than 0.5 °C, unfortunately, the establishment of fluorescence lifetime imaging microscopy as a routine method in biomedical laboratories will still require solid funding, specially trained personnel and, as a consequence, years to be accomplished. The other problem frequently associated with the use of organic dyes is their potential photo- and cytotoxicity.

To solve the abovementioned problems a genetically encoded fluorescent biosensor would represent an ideal solution. A general approach to engineer such a construction requires the combination of functional (temperature sensitive) and reporting (fluorescent) modules in one fusion protein. This approach has been successfully used to develop numerous chimeric sensor constructs. For example, the so-called chameleons (Camelion) are now widely used for research and diagnostic purposes^10^. Here, calmodulin (CaM) fused N- and C-terminally to fluorescent proteins forming a FRET pair, changes its conformation upon binding of Ca^2+^ ions, which affects the efficiency of excitation energy transfer between the two fluorescent proteins. Thus, changes in sensitized emission of the energy acceptor correspond to the changes of local Ca^2+^ concentration in the cell or in certain cellular compartments.

In this work we present our attempt to use the Orange Carotenoid Protein (OCP) as a functional part of a genetically encoded temperature sensor. OCP is a small 35-kDa water-soluble protein responsible for photoprotection in cyanobacteria^11–20^. OCP consists of two structural domains harboring a single ketocarotenoid molecule^21^. It is now well established that during the photoconversion of OCP by blue-green light and transition from the orange, OCP^O^ , to red, OCP^R^, form, the distance between the carotenoid and the C-terminal domain (CTD) of the protein significantly increases due to carotenoid translocation into the N-terminal domain (NTD) and disruption of protein-protein interactions in the inter-domain interface region^14,22,23^. Previously, using intrinsic and extrinsic fluorescent labels (either covalently and non-covalently bound to the protein structure) we have shown that changes in the geometry of OCP during the photocycle can be detected using steady-state and time-resolved fluorescence spectroscopy^15,24,25^. Since the rate constants of photoconversion and relaxation of OCP strongly depend on temperature, we conceived to use this protein as a functional module of a temperature sensor. Due to the high activation energy of OCP relaxation (~32 kcal/mol), fluorescence readout of such an OCP-based sensor may potentially provide an accuracy of temperature sensing better than 0.1 °C. Here we show that photoactive chimeric constructions based on OCP and fluorescent proteins are sensitive to temperature *in vitro* and discuss how this sensor could be further improved for cellular applications.

## Materials and Methods

### Cloning, protein expression, and purification

**Figure 1:**
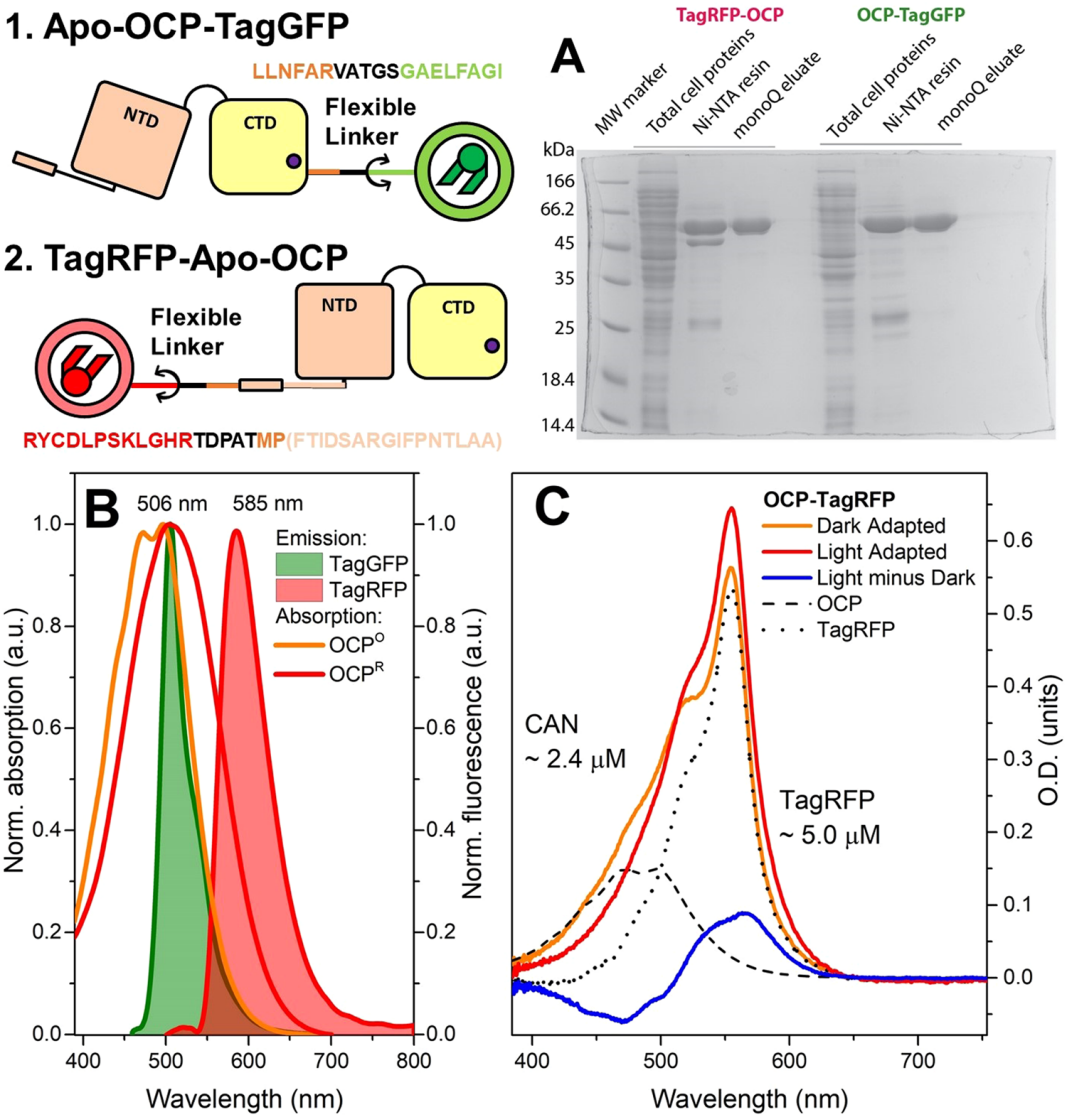
Top row, left: schematic representation of chimeric constructions. TagGFP and TagRFP are shown in green and red, respectively. NTD and CTD of OCP are shown in salmon and yellow. The linker introduced by cDNA cloning is present in black. The amino acid sequences of the flexible parts neighboring the introduced linker (black) are also indicated by color. Note that unfolding of the N-terminal extension of OCP (NTE) upon photoconversion^41^ may increase the length of the flexible linker region between the proteins. (**A**) SDS-PAGE of OCP-TagGFP and TagRFP-OCP chimeras at different stages of purification. (**B**) Normalized absorption spectra of OCP in the dark-adapted orange and photoactivated red state and normalized fluorescence spectra of OCP-TagGFP and TagRFP-OCP chimeras. Note the overlap between the carotenoid-based OCP absorption and the emission spectra of both fluorescent proteins. (**C**) Absorption spectra of the TagRFP-OCP chimera before (dotted line) and after the carotenoid incorporation (orange line), and upon photoactivation by actinic light. Numbers indicate TagRFP and carotenoid concentrations after overnight incubation of TagRFP-OCP apoprotein in the presence of COCP holoprotein. Estimations of concentrations were obtained considering identical molar extinction coefficients in fusion and individual proteins.

The nucleotide sequences coding for Red Fluorescent Protein (TagRFP) and Green Fluorescent Protein (TagGFP) were PCR-amplified from the Casper3-GR vector (Evrogen, Russia). The PCR product of TagRFP was digested by *BamHI* (New England Biolabs, USA) enzyme and cloned into the pQE81L-OCP expression vector^26^. The PCR product of TagGFP was utilized for overlapping PCR with the amplified sequence of *Synechocystis* OCP; the resulting OCP-TagGFP PCR product was cloned into the pQE81L expression vector utilizing *BamHI* and *NotI* restriction sites (New England Biolabs, USA). The 6xHis-tag derived from the pQE81L vector construct was identical in both types of chimeras. To produce chimeric proteins, SHuffle® T7 Competent *E. coli* (NEB, USA) cells were transformed by the resultant pQE81L-TagRFP-OCP and pQE81L-OCP-TagGFP plasmid constructs and incubated in LB medium overnight at 37 °C. At an OD_550_ of 0.6, protein expression was induced with 1 mM IPTG and continued for 6 h at 30 °C. All proteins were purified by immobilized metal-affinity and anion exchange chromatography to electrophoretic homogeneity and stored at 4 °C in the presence of 2 mM sodium azide. A schematic representation of both chimeric constructions is shown in Fig. 1. In order to obtain both holoforms of the chimeric constructions, first with TagRFP at the N-terminus of OCP (TagRFP-OCP) and second with TagGFP at the C-terminus of OCP (OCP-TagGFP) by carotenoid transfer in solution, recombinant proteins were co-incubated with carotenoid-(canthaxanthin, CAN)-binding OCP-CTD homodimers (COCP, C-terminal OCP-related Carotenoid Protein) as described previously^27,28^. After this incubation, samples were loaded on a MonoQ column (GE Healthcare, UK). Fractions from anion exchange chromatography were subjected to SDS-PAGE and visualized by Coomassie staining (Fig. 1A).

### Absorption measurements

Absorption spectra were recorded using a Perkin Elmer (Waltham, MA) Lambda-25 spectrophotometer. In all experiments, an M455L3 (Thorlabs, Newton, NJ) 900 mW light-emitting diode (LED) with maximum emission at 455 nm was used for blue-green illumination of the samples (actinic light for OCP^O^  → OCP^R^ photoconversion).

### Steady-state fluorescence measurements

The steady-state fluorescence measurements were performed using a FluoroMax-4 spectrofluorimeter (Horiba Jobin Yvon, Kyoto, Japan). Samples were diluted to OD ~0.01 units at 510 nm to avoid inner filter effects and reabsorption. Fluorescence emission of TagGFP and TagRFP was measured at excitation wavelengths set to 450 and 510 nm, respectively. The temperature of the sample was stabilized by a Peltier-controlled cuvette holder Qpod 2e (Quantum Northwest, Liberty Lake, WA).

### Picosecond time-resolved fluorescence measurements

Fluorescence-decay kinetics with picosecond time resolution was collected by a time- and wavelength-correlated single-photon counting setup (Becker and Hickl, Berlin, Germany). Excitation of TagGFP and TagRFP was performed with ps-pulsed lasers at 450 and 510 nm (InTop, Russia), driven at a repetition rate of up to 25 MHz. A set of longpass filters (Thorlabs) was used to block excitation light. Fluorescence decay curves were approximated by a sum of exponential decay functions with the SPCImage (Becker and Hickl, Germany) software package.

### Protein-protein docking

For the prediction of tentative TagRFP-OCP and OCP-TagGFP structures, protein docking was used. As a reference, the following atomic structures were used from the Protein Data Bank: 3MG1 (for OCP, NTD, CTD models)^29^, 3M22 (TagRFP)^30^, and 4XB4 (OCP-NTD)^14^. The full-atom structural models of linker conformations were calculated with the I-TASSER web-server^31,32^. Protein-protein rigid-body docking was performed with the HEX 8.0.0 package^33^. Macro-sampling (50 × 50) of starting donor-acceptor orientations was performed. To create a set of docking solutions, three different scoring functions were applied: Shape Only, Shape + Electro, Shape + Electro/OPLS Minimization. The top 990 unique docking poses (top 330 for each scoring function) were analyzed. Poses for TagRFP-OCP and OCP-TagGFP groups were selected according to the estimated linker constraints.

All experiments were conducted at least three times using different protein preparations.

## Results

In order to estimate how the difference in the overlap between the emission spectrum of the energy donor (Fluorescent Protein, FP) and the absorption spectrum of the acceptor (the carotenoid in OCP) affects excitation energy transfer (EET) in chimeric structures based on OCP, we compared two fluorescent proteins with distinct spectral properties as fusion partners of OCP – TagGFP and TagRFP. These FPs are characterized by high fluorescence quantum yield, are monomeric and very photostable^34^ and, therefore, are reasonable fusion partners for OCP. We have tried both, N- and C-terminal position of the fluorescent protein relative to OCP to find an optimal combination. After purification of the OCP-TagGFP and TagRFP-OCP chimeras expressed in regular *E. coli* strains (not producing ketocarotenoids, resulting in OCP apoprotein forms, Fig. 1), the visible absorption spectra of the samples were characteristic for the TagGFP and TagRFP chromophores, respectively, which proves  that fusion to OCP does not affect formation and maturation of the FP chromophore. In order to obtain the carotenoid-containing forms of the chimeras, we used a recently developed approach based on carotenoid delivery by carotenoid-binding protein COCP^27,28^. COCP exclusively binds canthaxanthin (CAN) as a chromophore, when expressed in appropriate carotenoid-producing *E. coli* strains^28^. Upon mixing of solutions of the apo-forms of the chimeras and the CAN-containing holo-COCP, we observed a gradual decrease of COCP absorption in the red region (550 nm) of the spectrum, accompanied by an increase of the absorption in the blue-green region (around 500 nm), which corresponds to carotenoid translocation and appearance of the orange form of OCP. This observation indicates that fusion of FPs to OCP does not prevent proper carotenoid binding by the OCP module, which is crucial for its photoactivity. Similar effects have been previously described in detail for interactions of COCP and apo-OCP^27^. Here, we must note that mixing of apo-forms of the chimeras and COCP in 1:1 concentration ratios resulted in about 50% efficiency of carotenoid transfer (see Fig. 1B), which could be explained by an equilibrium between the carotenoid transfer from COCP into the chimera and the reverse process. OCP-CTD-like forms were recently shown to be able to extract carotenoids from OCP in a state with separated domains^35^, so the reverse transfer of CAN is possible if the OCP part of the chimera is not always in a compact state. A goal for further improvements of OCP-based photoswitches is to improve the stability of the compact state in order to increase the efficiency of carotenoid delivery. On the other hand, improvement could be achieved by decreasing the stability of the carotenoid carrier system, which delivers the carotenoid to OCP.

**Figure 2:**
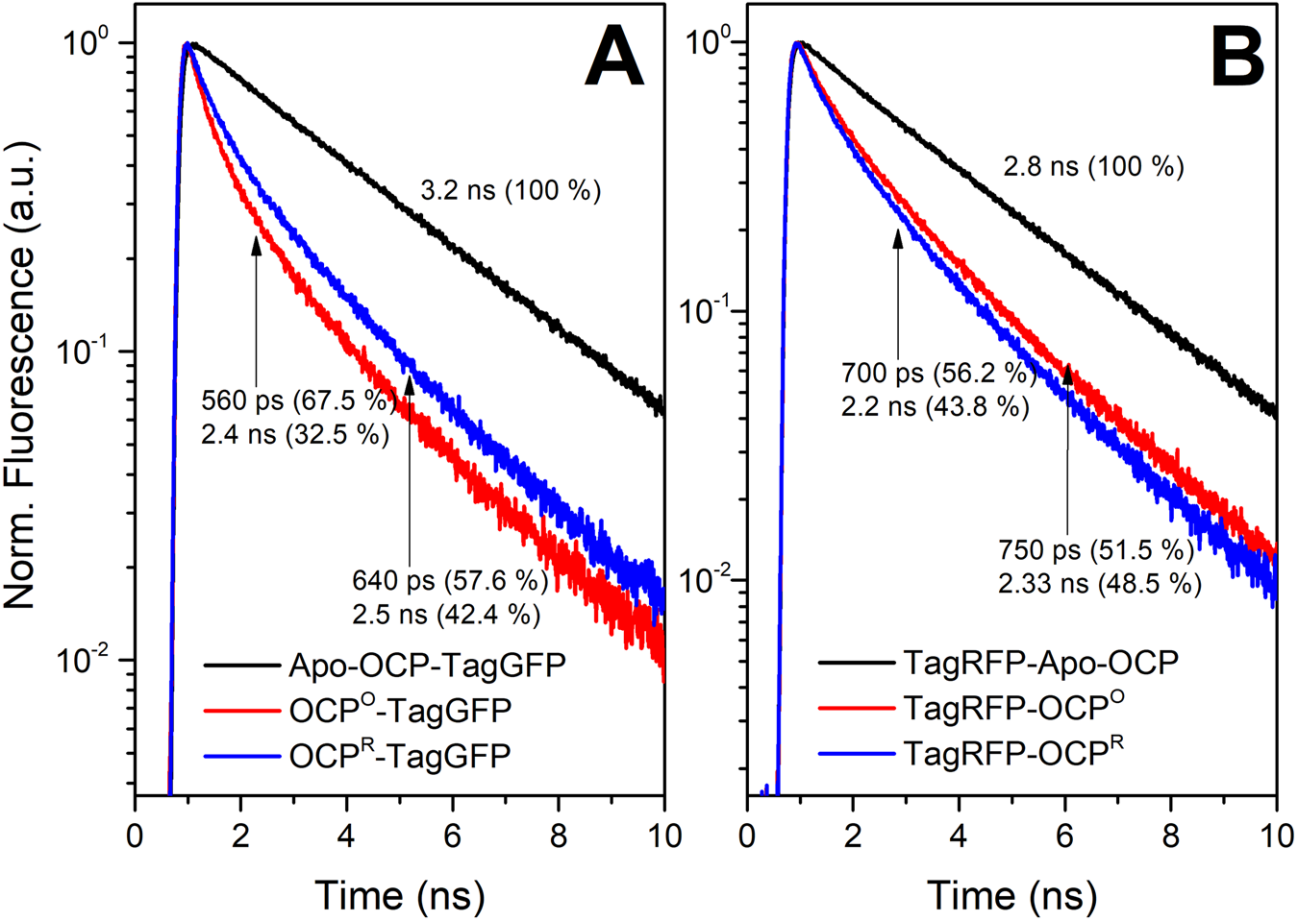
Fluorescence decay kinetics of the OCP-TagGFP (**A**) and TagRFP-OCP (**B**) chimeras in their apo-forms lacking a carotenoid molecule as an energy acceptor (black curve), with carotenoid (red) and after photoactivation of the OCP component (blue) by a 200 mW blue LED. Experiments were conducted at 5 °C in order to reduce the photoconversion rates^15^. Numbers indicate characteristic lifetimes and the corresponding amplitude contributions derived from the fitting of the decay curves by two exponential functions. The absence of decay components with lifetimes characteristic for the apoforms of the chimeras in the fluorescence decays of the holoforms indicates that the chimeras were fully loaded with carotenoids upon overnight incubation of the apo-forms with an excess of COCP as a carotenoid donor.

Upon illumination of the apoforms of OCP-TagGFP and TagRFP-OCP chimeras by actinic blue light (200 mW LED with maximum emission at 450 nm), we observed no changes of TagGFP or TagRFP absorption and no significant temporal changes in fluorescence intensity of the corresponding proteins, which indicates that the fluorescent proteins on their own remain photostable. After carotenoid incorporation into the chimeras, actinic blue light caused characteristic and reversible changes of carotenoid absorption (Fig. 2B) indicating that the presence of the fluorescent protein as part of an OCP-based chimera (i) does not prevent photoactivation of OCP and (ii) allows relaxation of the red OCP state. Even more important is the fact that the emission of the fluorescent proteins was sensitive to the photocyclic transitions of OCP.

Photoactivation of OCP as part of the chimera with TagGFP caused an increase of fluorescence intensity and average lifetime of TagGFP (Fig. 2A). In contrast, the same treatment caused an opposite effect in the TagRFP-OCP chimera, in which fluorescence was quenched upon OCP photoactivation. It should be noted that overall changes of fluorescent protein emission upon OCP photoactivation are relatively small compared to fluorescence quenching which occurs upon delivery of the carotenoid into the chimeras (Fig. 2). These observations indicate that (i) the position of carotenoid in a complex determines the efficiency of EET and (ii) the efficiency of EET from the fluorescent proteins to the carotenoid is high in both states of OCP, red and orange. Since we know that, upon OCP photoactivation, the carotenoid moves about 12 Å deeper into the NTD^14^, the observed changes in the quantum yields of energy donors could probably be explained by changes of the distance between the donor and acceptor of energy. However, examination of other factors affecting EET efficiency is needed.

First of all, the overlap integral between the emission spectra of the donor of energy and absorption of the acceptor must be taken into account. From the spectra in Fig. 1B it is clear that emission of TagGFP perfectly overlaps with the S_0_-S_2_ absorption of the carotenoid in OCP (in both OCP^O^  and OCP^R^ states), while TagRFP has a much lower overlap (see Fig. 1), which is in a good agreement with the observed difference in quenching of FP fluorescence (Fig. 2) after incorporation of CAN into OCP. Since OCP photoactivation causes a red shift of carotenoid absorption, the overlap with TagRFP emission increases (see Table 1, Förster radius *R*_0_^36,37^ increases from 40.9 to 47.9 Å, considering a random orientation of transition dipole moments), while it stays almost constant for the TagGFP-based chimera (*R*_0_ decreases from 57.3 to 55.1 Å), consistent with the notion that the overlap of the TagGFP emission spectrum with the OCP absorption spectrum almost does not change upon OCP photoactivation (Fig. 1B). It is important to note here that, in theory, the symmetry of the carotenoid responsible for the optically forbidden S_0_-S_1_ excitation of carotenoid could be violated due to conformational mobility, and, thus, energy transfer from the excited FPs might also occur via the S_1_ level of the carotenoid^38^. Another factor is the orientation of the transition dipoles. OCP^R^ shows characteristics of a molten globule^15,23^, in which CTD and NTD can move freely, and, thus, the assumption of the random orientation of the transition dipoles is reasonable. However, when OCP is in the compact orange state, this assumption may no longer hold. Further, the mutual mobility of a compact orange OCP and the FPs requires more careful consideration, since the fluorescence decay curves of both FPs are clearly biexponential, which indicates heterogeneity of the samples and requires further exploration.

**Table 1:** Excitation Energy Transfer from FPs to the carotenoid of OCP and calculated FRET parameters.

OCP State	Energy Donor	TagGFP		TagRFP	
	FP-OCP Complex Configuration	1 – “tight’’	2 – “loose”	1 – “tight	2 – “loose”
OCP^O^	EET Efficiency E, %	82.7 ± 1.5	25.9 ± 1.6	72.8 ± 1.3	15.5 ± 1.5
	Population, %	67.5 ± 2.7	32.5 ± 2.6	51.5 ± 2.4	48.5 ± 2.3
	Donor-Acceptor Distance *R*, Å	44.1	68.2	34.7	54.4
	Förster radius *R*_0_, Å	57. 3		40.9	
OCP^R^	EET Efficiency *E*, %	80.2 ± 2.4	22.8 ± 2.2	74.6 ± 2.9	20.0 ± 2.6
	Population, %	57.6 ± 3.0	42.4 ± 2.9	56.2 ± 2.8	43.8 ± 2.8
	Donor-Acceptor Distance *R*, Å	43.6	67.5	40.0	60.3
	Förster radius *R*_0_, Å	55.1		47.9	

EET efficiency values *E* and population fractions were estimated from the fluorescence decay components of FPs (see Fig. 2). Förster radius *R*_0_, and Donor-Acceptor distance *R* were calculated assuming the random orientation of the transition dipoles *κ*^2^.

**Figure 3:**
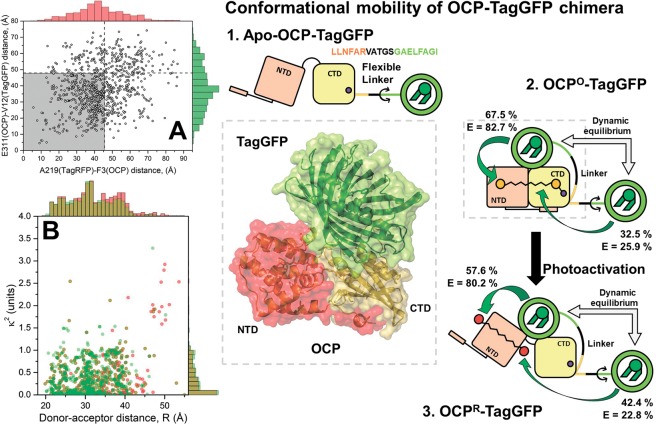
Left column – results of protein docking: (**A**) Distribution of distances between the first amino acids which are part of the fused proteins’ secondary structures: A219(TagRFP)-F3(OCP) and E311(OCP)-V12(TagGFP). Vertical and horizontal lines separate docking solutions available for TagRFP-OCP and OCP-TagGFP, respectively, based on estimations of the linker length and neighboring non-folded stretches of amino acids. (**B**) Distribution of distances (*R*) between the FPs’ chromophores and the carotenoid of OCP and orientation factors (*κ*^2^) for chromophores among each group of docking solutions. Right – a working model of FP–OCP interactions in chimeric structures for the example of OCP-TagGFP. Due to the flexibility of the linker connecting the rigid parts of OCP and TagGFP, the chimera’s structure can adopt multiple conformations. However, in the absence of the carotenoid (i.e. in Apo-OCP-TagGFP), this does not affect the emission of TagGFP. Carotenoid incorporation then leads to the formation of the compact OCP° state, in which TagGFP emission is quenched due to EET. However, the conformational mobility of TagGFP in relation to OCP leads to a heterogeneity of EET efficiencies. Analysis of the TagGFP fluorescence decay in the presence of the energy acceptor (see Fig. 2) shows that two distinct populations of chimeras are present. Numbers indicate the yield of each population and the corresponding efficiencies (*E*) of EET. Photoactivation of OCP affects the dynamic equilibrium between the conformers.

An element influencing the mutual orientation of OCP and FP in the complex is the length of the linker. Whereas a short linker should yield a relatively rigid complex with a limited number of possible configurations, a long and unstructured linker should entail larger conformational mobility of the complex. To estimate the mutual mobility of OCP and FP in the chimeric structure, not only the five amino acid-long linker, but also all neighboring amino acids potentially assuming an unfolded secondary structure are of interest, which are: RYCDLPSKLGHRTDPATMP for TagRFP-OCP, and LLNFARVATGSGAELFAGI for OCP-TagGFP (the linkers are underlined in both cases). These sequences, which provide for the connection between the amino acids A219(TagRFP)-F3(OCP) and E311(OCP)-V12(TagGFP) are likely to be non-folded, solvent accessible and flexible. The estimated maximum lengths of such flexible linkers in a fully extended conformation are 46 and 48 Å for TagRFP-OCP and OCP-TagGFP, respectively, as inferred from I-TASSER modeling. In the following, we used these linker length estimations as restraints for predicting possible poses by protein docking algorithms (Fig. 3A, see Materials and methods section for details). Further, we calculated the distance between the geometric centers of carotenoid and the chromophores of the fluorescence proteins in all possible TagRFP-OCP and OCP-TagGFP docking solutions, which was estimated to be equal to 32 ± 6 and 31 ± 6 Å, respectively (Fig. 3B). The performed protein docking simulation of chimeric structures led to several results: (i) no specifically oriented dispersion complexes of OCP and FPs were found, which could argue that there are no specific interaction sites on these proteins and (ii) average values of the inter-pigments interaction parameters were calculated. Further, one can speculate that the “stuck together” complexes of OCP and FPs, which are represented by the sets of docking solutions, are one of two distinct fractions observed in fluorescence decay measurements (Fig. 2). The second fraction refers to the “free” complexes, in which both counterparts avoid dispersion interactions with each other and are surrounded by the solvent (Fig. 3). In such “free” cases, the protein chromophores are separated by rather significant distances and the assumption about the random orientation of the transition dipoles is justified. The coexistence of these two groups of configurations explains the biexponential decay of FP fluorescence in the presence of the OCP energy acceptor. Thus, the fast component of the fluorescence decay (Fig. 2) can be assigned to structures formed by non-specific interactions between protein interfaces resulting in high EET efficiency, and the slow component is due to a less efficient energy transfer from FP chromophores to the OCP carotenoid when separated by a flexible linker (Table 1). Calculations show that in TagRFP-OCP, the characteristic distances between the RFP chromophore and the OCP carotenoid are equal to 34.7 and 54.4 Å, considering the random orientation of transition dipoles. Thus, both distances obtained from the experiment are in reasonable agreement with our *in silico* estimations of possible complex geometries and linker length. However, analyzing the fluorescence decay of OCP-TagGFP we found characteristic distances equal to 44.1 and 68.2 Å. Since the structures of TagGFP and TagRFP are very similar, it seems unlikely that distances between the donor and acceptor of energy in a compact “stuck together” complex with OCP are so dramatically different. We assume that in case of OCP-TagGFP the reason of distance overestimation is related to changed conditions regarding the relative orientation of the transition dipoles, which is expressed by the factor in the Förster formulation of FRET. Considering that distance between the donor and acceptor of energy in OCP-TagGFP should be nearly the same as in TagRFP-OCP, we can estimate the value of *κ*^2^ corresponding to the EET efficiency observed experimentally. Considering characteristic donor-acceptor distance ca. 32 Å, we found the corresponding value of *κ*^2^ to be equal to 0.082, which is a result of an almost perpendicular orientation of the transition dipoles. Indeed, the obtained distribution of *κ*^2^ values on the basis of protein docking solutions shows an abundance of such complexes suggesting this geometry. Further, we used a combination of *κ*^2^ and *r* values as constraints to identify potential structure(s) of OCP-TagGFP chimeras among the results of protein docking. It appeared that from 990 poses suggested by protein docking, we were able to find few which correspond to such a combination of FRET parameters. An example of such structures is shown in Fig. 3. As mentioned before, we tend to exclude specific interactions between OCP and FPs, thus structural model presented in Fig. 3 should not be considered as one and the only option.

Thus, we assign the two fluorescence decay components to distinct groups of structures corresponding to a multitude of non-specific interactions between FP and OCP connected by the flexible linker, which result in different EET efficiency in these subpopulations. It is important to note that upon the photoactivation of OCP in both studied systems, we observed changes predominantly in amplitudes of the fast component, which is assigned to tightly interacting OCP and FP, while the corresponding lifetimes remained almost constant. We assume that such a transition could be enforced by OCP photoactivation, since this process increases the conformational mobility of OCP itself^15,39^. Thus, in the photoactivated state, we must not only consider possible motions of the FP module but also mutual motions of the OCP domains. In this regard, we assume the orientation of the transition dipoles as random for the photoactivated state.

In summary, two types of fluorescent reporter proteins consisting of an FP fused to either the N- or the C-terminal end of the two-domain photoactive OCP were obtained, the fluorescence of which was shown to be sensitive to the photocycle of OCP. It is important to note that changes of the overlap integral between the TagRFP emission and OCP absorption spectrum are large enough to compensate for the increase of the distance between the donor and acceptor (Table 1). Adjustments of this factor could be further utilized if one would use near-infrared emitting FPs^40^ (at the N-terminal domain of OCP). In such systems, we expect very low EET efficiency in the orange state, which will increase upon OCP photoactivation. This is also important because probing fluorescence of such near-infrared (or far-red) fluorescent proteins will not trigger photoactivation of OCP due to the low EET efficiency in the orange state, thus, the contrast between the states of the sensor could be significantly improved further.

However, the position of the reporter module regarding the N- or C-terminus of OCP is also very important from the perspective of photocycling. We found that, under similar experimental conditions, the relaxation rates of the photoactivated chimeras are different. After photoactivation, TagRFP-OCP undergoes significantly slower relaxation to the initial state compared to OCP-TagGFP. This observation could be explained considering the sequence of protein reorganization steps, which occur during OCP relaxation. Detachment and unfolding of the short αA helix of the N-terminal extension, NTE, was reported upon photoactivation of OCP^22,41,42^. Unfolding of the NTE may largely increase the length of the flexible linker (from 46 to 110 Å, see Fig. 1). In the dark-adapted state, the NTE is connected to a specific site on the OCP-CTD^14,21,29^; thus, the back relaxation process requires refolding of the helix and reestablishment of protein-protein interactions. It is very reasonable to assume that the 27 kDa TagRFP connected to the NTE may slow down this process. In contrast, since it is known that translocation of the carotenoid from the NTD back into the CTD occurs faster than the relaxation of the protein^25^, fast relaxation of OCP-TagGFP would be expected. Such a dependence of the relaxation rate on the position of the reporter module in the structure of the chimera provides an opportunity to select between fast and slow responding temperature sensors.

**Figure 4:**
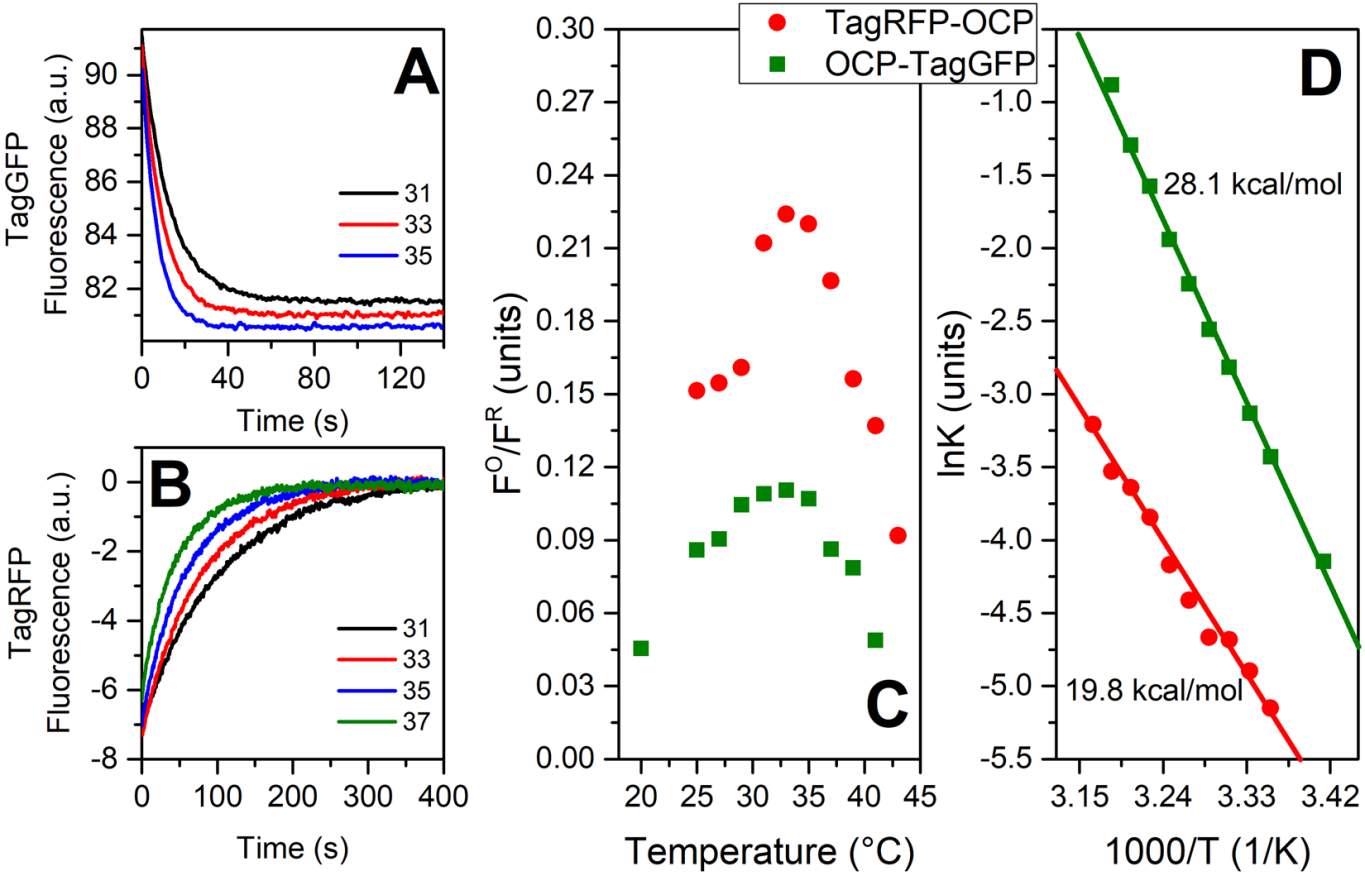
Characteristic time-courses of fluorescence intensity of the OCP-TagGFP (**A**) and TagRFP-OCP (**B**) chimeras, measured after photoactivation of the OCP component by a 200 mW blue LED at different temperatures (as indicated by different line colors). (**C**) Relative contrast between the fluorescence intensity of OCP-TagGFP and TagRFP-OCP chimeras in photoactivated (F^R^) and dark-adapted (F^O^) states. Arrhenius plots of relaxation rates of OCP-TagGFP and TagRFP-OCP and corresponding energy barriers.

Relaxation of the photoactivated chimeras accelerates with increasing temperature (see Fig. 4). In the case of OCP-TagGFP, we observed an activation energy (E_a_) barrier equal to 28.1 kcal/mol, which is close to the value reported for OCP (~32 kcal/mol^17^), while the relaxation of TagRFP-OCP fluorescence is less temperature-dependent (19.8 kcal/mol). Such high E_a_ values imply that a temperature increase by 1 °C results in a ~15% increase of the rate constant. Thus, it is rather easy to detect even minute temperature changes with sufficient accuracy (down to 0.1 °C), as desired and adequate for intracellular temperature imaging.

We must note that the contrast between the states decreases with decreasing temperature (Fig. 4B), which is not typical for isolated OCP (see Fig. 1D in^15^). This effect could be explained by the photoactivation of OCP due to EET, which inevitably occurs during the probing of FP fluorescence. This observation suggests that OCP-based sensors in conjunction with far-red or infrared fluorescent protein as a reporter module may provide better contrast in a wider range of temperatures if the values of the overlap integral and, therefore, the efficiency of EET in the orange state are sufficiently low.

Considering the characteristic relaxation rates (see Fig. 4), a typical experiment to determine the temperature in the 30–40 °C range will require about 60 s for the C-terminally located reporter (OCP-TagGFP chimera) and 300 s for the FP fused to the N-terminus of OCP (TagRFP-OCP chimera). Both timescales are reasonable for experiments in which the object of interest does not move (cells attached to a surface, etc.) and requires measurements of the fluorescence intensity rather than lifetimes used in other works and requiring much more sophisticated equipment. In cases when such long exposures are impossible, we assume that the temperature could still be estimated based upon the fast relaxation of an intermediate red OCP state, OCP^RI^, the characteristic lifetime of which at 30 °C is as fast as 300 µs^25^, which could be interpreted as an average time necessary for the carotenoid to restore contacts with Trp and Tyr residues. The activation energy for this elementary stage is similar to that of the relaxation of the red state with separated domains. Since this red intermediate is characterized by a red-shifted absorption, while still being in a compact protein state, a “fast” fluorescent sensor should be optimized for the changes of the overlap integral, but not for the distances between the donor and acceptor. Although recording a sequence of images at such rates is technically difficult, requiring excitation of the OCP-based sensor with short light pulses, the presented sensor concept could potentially provide information even about fast intracellular processes. Another opportunity to increase the rate of OCP-based temperature measurements is to use homologues of this protein like members of the so-called OCP2 clade, which are present in certain types of cyanobacteria and characterized by a significantly faster relaxation of the red state^43^, compared to the one of OCP from *Synechocystis* used in this work. Alternatively, point mutations in *Synechocystis* OCP can also be used to adjust the rates of OCP photoactivation and relaxation and, therefore, to improve the applicability of the OCP-based sensors further.

## Conclusion

In this work, we present a novel approach to construct a genetically encoded temperature sensor based on the photoactive OCP fusions with the GFP-like fluorescent proteins of two types. In addition, we describe its properties *in vitro*, providing a platform for further optimization of the sensor before proceeding with *in vivo* experiments. We found that both systems are potentially suitable for temperature measurements with high precision by following the time-course of fluorescence intensity after photoactivation of the OCP component. Although both types of sensors work properly showing proof-of-concept, their functionality could be further improved considering the following facts: (1) The position of the fluorescent protein affects the rate of relaxation, making it slow in the case of FP fusion to the N-terminus of OCP, probably due to slow interactions of the OCP-NTE with the OCP-CTD due to NTE refolding; (2) the overlap integral between the emission spectrum of the FP and absorption of the carotenoid determines the efficiency of EET; (3) EET results in (partial) photoactivation of OCP; (4) structural heterogeneity of the chimeric constructions is affected by photoactivation of the OCP component. Based on these facts, we can propose several means to optimize the OCP-based chimeras for temperature sensing in the future. First, it is important to reduce the EET efficiency in the dark-adapted state in order to prevent OCP photoactivation during probing the FP emission. This could be achieved by minimization of the overlap integral between the absorption of the orange OCP form and the emission of some far-red or infrared FP (such as iRFPs^44^). Second, to increase the contrast between the states in the photoactivated form of the chimera, the FP must be as close to the NTD as possible. This means that the FP must be located immediately at the N-terminus of OCP, with a minimal linker. Since OCP could still be photoactive even in the absence of NTE^42^, we assume that it may be deleted in order to put the reporter fluorescent module closer to the energy acceptor. Alternatively, the flexible FP-derived part of the linker may also be shortened. After optimization of the chimera and introduction of protein parts for the selective delivery into specific compartments of the cell, one may use OCP-based chimeras to measure organelle- and compartment-specific intracellular temperature.
